# Effects of regional brain volumes on cognition in sickle cell anemia: A developmental perspective

**DOI:** 10.3389/fneur.2023.1101223

**Published:** 2023-02-13

**Authors:** Shifa Hamdule, Melanie Kölbel, Hanne Stotesbury, Russell Murdoch, Jonathan D. Clayden, Sati Sahota, Anna Marie Hood, Christopher A. Clark, Fenella Jane Kirkham

**Affiliations:** ^1^Developmental Neurosciences Section, UCL Great Ormond Street Institute of Child Health, London, United Kingdom; ^2^Sleep Education and Research Laboratory, UCL Institute of Education, London, United Kingdom; ^3^Department of Medical Physics and Biomedical Engineering, University College London, London, United Kingdom; ^4^Division of Psychology and Mental Health, Manchester Centre for Health Psychology, University of Manchester, Manchester, United Kingdom; ^5^Clinical and Experimental Sciences, University of Southampton, Southampton, United Kingdom

**Keywords:** sickle cell disease, brain volume, cognition, development, working memory, processing speed

## Abstract

**Background and objectives:**

Cognitive difficulties in people with sickle cell anemia (SCA) are related to lower processing speed index (PSI) and working memory index (WMI). However, risk factors are poorly understood so preventative strategies have not been explored. Brain volumes, specifically white matter volumes (WMV) which increases through early adulthood, have been associated with better cognition in healthy typically developing individuals. In patients with SCA, the reduced WMV and total subcortical volumes noted could explain cognitive deficits. We therefore examined developmental trajectories for regional brain volumes and cognitive endpoints in patients with SCA.

**Methods:**

Data from two cohorts, the Sleep and Asthma Cohort and Prevention of Morbidity in SCA, were available. MRI data included T1-weighted axial images, pre-processed before regional volumes were extracted using Free-surfer. PSI and WMI from the Weschler scales of intelligence were used to test neurocognitive performance. Hemoglobin, oxygen saturation, hydroxyurea treatment and socioeconomic status from education deciles were available.

**Results:**

One hundred and twenty nine patients (66 male) and 50 controls (21 male) aged 8–64 years were included. Brain volumes did not significantly differ between patients and controls. Compared with controls, PSI and WMI were significantly lower in patients with SCA, predicted by increasing age and male sex, with lower hemoglobin in the model for PSI but no effect of hydroxyurea treatment. In male patients with SCA only, WMV, age and socioeconomic status predicted PSI, while total subcortical volumes predicted WMI. Age positively and significantly predicted WMV in the whole group (patients + controls). There was a trend for age to negatively predict PSI in the whole group. For total subcortical volume and WMI, age predicted decrease only in the patient group. Developmental trajectory analysis revealed that PSI only was significantly delayed in patients at 8 years of age; the rate of development for the cognitive and brain volume data did not differ significantly from controls.

**Discussion:**

Increasing age and male sex negatively impact cognition in SCA, with processing speed, also predicted by hemoglobin, delayed by mid childhood. Associations with brain volumes were seen in males with SCA. Brain endpoints, calibrated against large control datasets, should be considered for randomized treatment trials.

## 1. Introduction

Sickle cell anemia (SCA) is an inherited single gene disorder affecting millions of people worldwide (1). Cognitive difficulties in SCA occur regardless of presence of stroke or silent cerebral infarction (SCI) (2). Commonly reported cognitive deficits include reduced processing speed and lower working memory (3–6) which may affect quality of life (7). There is a lack of literature investigating risk factors for cognitive difficulties over time in the SCA population. Reduced white matter volumes (WMV) and subcortical volumes have been found in both adults and children with SCA (8, 9) but any association with cognitive difficulties is under-studied in this population (10).

In healthy individuals, increase in WMV is associated with increase in general IQ (11). In young adult patients with SCA, regional white matter volumes show atrophy in frontal, temporal and parietal regions (12). Reduced IQ in patients with SCA appears to be in part related to reduced processing speed index (PSI) (13). Although PSI has been associated with white matter microstructural damage (13), there are few data examining any association between WMV and PSI, although one study reported reduced WMV associated with IQ loss in male patients with SCA (12). It is therefore reasonable to explore the possibility that reduced WMV in patients with SCA is associated with reduced PSI.

There are few studies on subcortical volumes and cognitive function in the general population although the available evidence suggests that an association after hypoxic-ischemic exposure but not in healthy children (14, 15). Total subcortical volumes are reduced in children with SCA (16) and smaller volumes have been noted in individual subcortical regions including the hippocampal subfields (17), amygdala, pallidum, caudate, putamen and thalamus in adults (9) and children with SCA (16). In adults with SCA, reduced basal ganglia and thalamus volumes were associated with lower working memory index (WMI) (9) but there are no equivalent data in children with SCA.

Studies looking at developmental trajectories of volumetric growth and cognitive development in SCA have reported mixed results. Longitudinal data studying cognitive development showed reduction in verbal IQ and coding, a measure of processing speed, over time in patients with SCA (18). A cross-sectional study plotting trajectories suggested reduced processing speed and working memory in children with SCA with increasing age (14) but there are few data examining any association with brain volume. A longitudinal study of children with SCA enrolled in the Silent Infarct Transfusion (SIT) trial showed reduction in global brain volume over time, but no association with change in IQ (19, 20). The cross-sectional data of Steen et al. (20) did not find any reduction in regional brain volumes but suggested maturational delay in gray matter volume growth in children with SCA but no differences in the WMV development between patients and controls. Chen et al. (21) on the other hand found that WMV in children with SCA was increasing at half the rate of controls (22). However, most studies looking at brain volume or cognitive developmental trajectories lacked control groups, were underpowered and reported failure to follow-up or mixed findings, warranting further investigation of brain volume and cognitive developmental trajectories in children with SCA (20).

In summary, research suggests that volumes of different brain regions might predict cognitive outcomes in children with SCA. Moreover, developmental trajectories may differ between patients with SCA and controls. Therefore, the purpose of this cross-sectional study was to examine the relationship between regional brain volumes (WMV and total subcortical volume) and cognitive variables (PSI and WMI). Hence, we hypothesized:

1. WMV would predict PSI in patients with SCA and controls.
2. Total subcortical volumes would predict WMI in patients with SCA and controls.
3. Sex differences would be observed in patients for WMV predicting PSI and total subcortical volumes predicting WMI.
4. Developmental trajectories for cognitive variables, as well as regional volumes, would differ between patients with SCA and controls.

## 2. Methods

### 2.1. Participants

Participants were enrolled in two studies (23): the Sleep Asthma cohort study (SAC) and the Prevention of Morbidity in SCA 2b (POMS) trial, and were aged >8 years at recruitment and assessment (2015–2019). Participants were ineligible if they were participating in blood transfusion or oxygen therapy trials, receiving nocturnal oxygen support, had chronic lung diseases (except asthma) or respiratory failure. Additional exclusion criteria for the POMS study included hospital admissions for acute sickle cell within 1 month of enrolment, more than 6 hospital admissions within 12 months of enrolment, mean overnight oxygen saturation of <90% for more than 30% total sleep time as measured on overnight oximetry, severe sleep apnea determined by 4% oxygen desaturation index > 15/h, and blood transfusion within 3 months of enrolment or chronic transfusion. The SAC study included patients regardless of sickle or sleep related morbidity or transfusion status. Healthy ethnically matched controls with no history of neurologic or psychiatric conditions were recruited from the community.

### 2.2. Ethical considerations

West London and South Yorkshire research ethics committees granted approval for the studies. Fully informed consent was obtained from adults and from parents/guardians with assent for children. Procedures were conducted in accordance with the Declaration of Helsinki.

### 2.3. Neurocognitive variables

The Weschler Scales were used to measure cognitive variables. For FSIQ, the Weschler Intelligence Scale for Children (WISC-IV) was used in SAC patients and controls under 16 years, the Weschler Adult Intelligence Scale (WAIS-IV) for adult SAC patients and controls, and the Weschler Abbreviated Scale of Intelligence (WASI-II) for POMS patients. The primary cognitive outcomes were PSI and WMI which were derived from the WISC-IV and WAIS-IV, with subtests administered separately for the POMS patients. PSI was calculated from Symbol Search and Coding subtests while WMI was calculated from the Arithmetic and Digit span subtests on the WISC-IV and WAIS-IV, respectively. There are strong correlations between editions of the Wechsler scales (WASI, WISC and WAIS) and between the adult and child versions (WISC and WAIS), justifying their inclusion in the same analyses (13, 24). Trained assessors double scored each test and were blinded to participant disease status (13). In case of disagreement or ambiguity, an independent assessor's opinion was sought.

### 2.4. Socioeconomic variables

Education deciles (25), obtained from UK postcodes, were used as an indicator of socioeconomic status (SES). Education deciles rank residential areas from 1 (most deprived) to 10 (least deprived) based on several indicators, including average scores for students aged 7–11 years and 14–16 years in state-funded schools, absence from secondary schooling, proportion of people studying beyond 16 years, entry into higher education, language proficiency and proportion of working adults with low qualifications.

### 2.5. Hematologic variables

Hemoglobin was acquired from patient medical records up to 6 months prior to the day of cognitive testing. Reference norms were used for controls (26). Oxygen saturation (SpO_2_) was measured using a pulse oximeter on the day of the cognitive testing for SAC participants and during baseline clinic visits for POMS patients. Medication history was also recorded. Since previous studies have shown an association between hydroxyurea use and cognitive functioning (27), hydroxyurea use was included in the models.

### 2.6. MRI acquisition

Within 2 weeks of cognitive assessments, all participants underwent scans on a 3T Siemens Prisma (Erlangen, Germany) MRI with 80 mT/m gradients and a 64-channel receive head coil. Axial T1 weighted images were acquired with repetition time (TR) = 2,300 milliseconds (ms), echo time (TE) = 2.74 ms, TI = 909 ms, flip angle = 8° and voxel size = 1 x 1 x 1 mm^3^.

The images were processed and analyzed using the “*recon-all*” pipeline in FreeSurfer version 4.5 (https://surfer.nmr.mgh.harvard.edu/) (28, 29). In summary, FreeSurfer conducts several pre-processing steps including motion correction, intensity normalization, affine transformation to the Montreal Neurological Institute (MNI) atlas, normalization to the original T1-weighted image, and skull stripping. For segmenting specific regional brain volumes, FreeSurfer uses the following steps: linear registration to Gaussian Classifier Atlas, canonical segmentation, and subcortical segmentation of individual regions (pallidum, caudate, putamen, thalamus, hippocampus, amygdala and accumbens).

### 2.7. Statistical analyses

**Figure 1:**
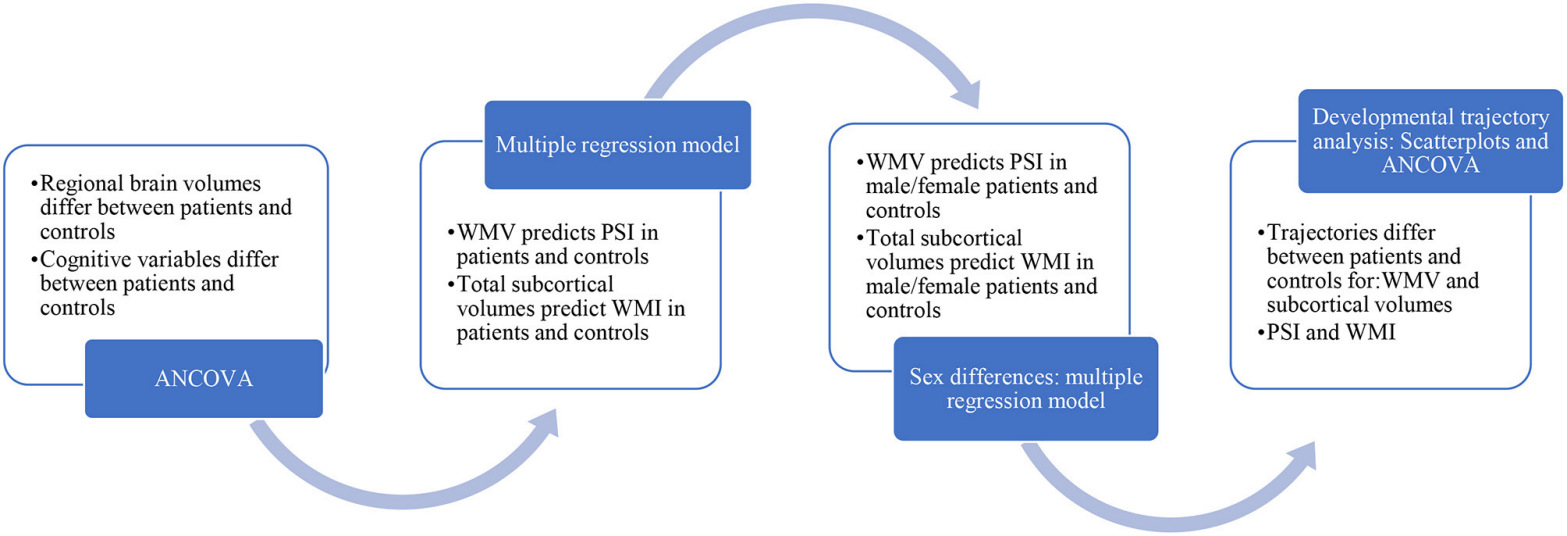
Statistical analysis.

IBM SPSS Statistics version 28.0.0.0 was used for statistical analyses. All variables were tested for normality using the Shapiro-Wilk and Levene's tests. ANCOVA was conducted to compare cognitive variables (PSI and WMI, adjusting for age, sex, and SES) and regional brain volumes [WMV and total subcortical volume, adjusting for age, sex, and intracranial volume (ICV)] between patients and controls. One patient with extremely high WMV (3SD above mean) was excluded from the analysis. Multiple linear regression models were generated for patients and controls, for WMV predicting PSI, and for total subcortical volumes predicting WMI. See Figure 1 for details.

Previous literature has reported an association between WMV and IQ only in male patients with SCA (12) which prompted us to look at sex differences in our sample. In an exploratory analysis, separate multiple linear regression models (WMV predicting PSI and total subcortical volumes predicting WMI) were generated for males and females for both groups-patients and controls. We also examined if there were differences in hemoglobin levels between male and female patients using an independent sample *t*-test. We wanted to examine if there were volumetric differences between patients and controls in the individual regions of the subcortex. Hence, an ANCOVA was used to compare volumes of individual regions of the subcortex (basal ganglia, thalamus, amygdala, and hippocampus) adding age, sex and ICV as covariates. Since total subcortical volumes were associated with WMI only in male patients with SCA, we wanted to investigate the effect of individual subcortical regions on WMI in male patients. Multiple linear regression models (adjusting for ICV and age) were generated for male patients for volumes of individual regions of the subcortex predicting WMI. Model fits were evaluated using Cook's distance and an analysis of residuals.

Developmental trajectories for cognitive variables and regional brain volumes (WMV and total subcortical volumes) were compared for patients and controls using the method outlined by Thomas et al. (30). In summary, linear regression models for cognitive variables and brain volumes were plotted by age for controls as well as patients to assess if there were any valid trajectory comparisons. For developmental trajectories to be valid, linear regression with age must be significant for the whole group (patients + controls) or it must be significant for typically developing controls. For valid trajectories (WMV and PSI), the two groups (patients and controls) were compared using analyses of covariances (ANCOVA) with regional brain volumes (adjusting for ICV and sex) or cognitive variables as response variable and age and group as covariates. An interaction term of age x group was also included in the analysis. Variables were fitted as a function of the following equation:


$$\begin{array}{l} \text{Volume}/\mathrm{cognitive}\;\mathrm{variable}=(\mathrm{Intercept}\;\mathrm{for}\;\text{group})\\ \;+(\text{Age}{\;}^{*}\;\text{gradient}) \end{array}$$

We examined the groups at onset at 8 years (intercept) and the slopes for rate of change in development between two groups.

Participants >30 years were excluded in the WMV trajectory analysis as, in typically-developing populations, WMV increases until the age of 28 years and then declines, creating a curved trajectory beyond that age (31).

## 3. Results

### 3.1. Demographic variables

Participant characteristics are described in Table 1. No significant differences were found between patients (*n* = 129) and controls (*n* = 50) regarding age, sex, and SES (*ps* > 0.05). The mean age of patients and controls was 19.18 years and 17.25 years, respectively. Sixty-six of 129 patients with SCA (51%) and 21 of 50 controls (were male. Mean hemoglobin and SpO_2_ levels for patients with SCA (88.13 g/l and 96.69%) were significantly lower than the reference norm for controls (134 g/l and 98.54%) (Table 1).

**Table 1:** Demographic details.

**Variables**	**SCA**									**Control**			**T score**	***P*** **-value**
Patient group	129									50 (33 siblings)			-	-
Male (%)	66 (51.1)									21 (42)			-	-
Genotype (SS, other)	125, 4									0, 50			-	-
	**Male**			**Female**			**Total**			**Total**				
	**Mean**	**SD**	**Std error**	**Mean**	**SD**	**Std error**	**Mean**	**SD**	**Std error**	**Mean**	**SD**	**Std error**		
Age	17.94	8.93	1.18	19.84	9.11	1.18	19.18	9.45	.84	17.25	8.44	1.19	1.255	0.276
SES	5.21	2.43	.322	5.27	1.91	.25	5.39	2.12	.213	4.92	2.00	.28	1.301	0.255
Hemoglobin (g/l)	88.97	15.20	2.01	88.14	13.65	1.77	88.13	15.03	1.32	134.0	8.17	1.15	−26.127	<.001
SpO_2_	96.47	2.62	.346	96.18	2.76	.360	96.69	2.682	.237	98.54	1.41	.221	−3.606	<.001

SES, socioeconomic status; SpO_2_, oxygen saturation from pulse oximetry.

### 3.2. MRI and cognitive variables

The data met the statistical assumptions for ANCOVA. The details are summarized in Table 2. WMV was lower in patients with SCA with a trend toward significance **(***p* = 0.057) while total subcortical volumes were lower in patients with SCA than controls but not statistically different (Table 2). PSI (mean difference = 7.03) and WMI (mean difference = 6.39) were significantly lower in patients than controls. There were no differences in WMV or total subcortical volumes between males and females in the patient or control group (Table 3). Hemoglobin was not significantly different between the sexes in the patient group (*p* = 0.6). In both groups, females had significantly higher PSI than males (mean difference in patient group = 7.18) (Table 3).

**Table 2:** Analysis of covariance (ANCOVA) for MRI and cognitive variable between SCA patients and controls.

**Variable**	**Adjusted mean**		***F*** **statistic (1,161)**	***p***
	**Patients**	**Controls**		
White matter volume (mm^3^)	408640.21	416643.93	3.690	0.057
Subcortical volume (mm^3^)	58709.31	58475.72	0.192	0.662
Processing speed index	88.79	95.82	10.881	**0.001**
Working memory index	91.79	98.18	7.981	**0.005**

Bold values signify statistical significance.

**Table 3:** ANCOVA for sex differences: Controls.

**Variable**	**Controls**				**Patients**			
	**Adjusted mean**		**F statistic (1, 46)**	***p***	**Adjusted mean**		***F*** **statistic (1, 128)**	***p***
	**Females**	**Males**			**Females**	**Males**		
WMV (mm^3^)	404128.76	451397.24	0.398	0.532	390482.61	421954.40	2.600	0.110
Subcortical Volume (mm^3^)	57542.85	61342.63	0.431	0.515	56884.11	60064.70	0.398	0.529
PSI	100.75	92.90	5.288	**0.026** ^b^	92.17	84.99	10.917	**0.001** ^c^
WMI	99.45	97.67	0.331	0.568	93.44	89.82	2.273	0.134

WMV, white matter volume; PSI, processing speed index; WMI, working memory index.

^a^*p* > 0.05 < 0.1;

b *p* < 0.05;

c *p* < 0.005.

Bold values signify statistical significance.

### 3.3. Associations between neurocognitive and MRI variables

Multiple regression analyses were conducted for patients and controls separately to predict PSI from WMV and WMI from total subcortical volumes. Both models were adjusted for age, sex, SES, ICV, hemoglobin, hydroxyurea use and SpO_2_. WMV was associated with PSI with a trend toward significance (*p* = 0.068; Table 4). Total subcortical volume was not significantly associated with WMI (*p* = 0.103; Table 5). Age and sex predicted both PSI and WMI in patients (Tables 4, 5). Hemoglobin was independently associated with PSI in patients (Table 4). In controls, sex predicted PSI but age and SES did not (Table 4) while SES predicted WMI but age and sex did not (Table 5). Hydroxyurea use was not associated with PSI (Table 4) or WMI (Table 5) in the patient group.

**Table 4:** Regression coefficients for processing speed index in patients.

	**Patients**			**Controls**		
**Variable**	**Unstandardised b**	**Standardized B**	***p***	**Unstandardised b**	**Standardized B**	***p***
WMV	6.843^E − 5^	**0.243**	0.068	−4.667E-5	−0.177	0.569
Hemoglobin	0.161	**0.178**	0.047	0.391	0.272	0.360
SpO_2_	0.224	0.523	0.602	1.882	0.221	0.289
Sex	−7.418	–**0.283**	0.011	−13.071	–**0.534**	**0.039**
Age	−0.469	–**0.345**	< 0.001	−0.256	−0.191	0.345
SES	0.817	0.136	0.126	0.342	0.059	0.712
ICV	−1.551^E − 5^	−0.150	0.359	1.872E-5	0.205	0.496
Hydroxyurea use	1.636	0.059	0.509	-	-	-

Adjusted *R*^2^, 0.156; WMV, white matter volume; SpO_2_, oxygen saturation from pulse oximetry; SES, socioeconomic status; ICV, intracranial volume.

Bold values signify statistical significance.

**Table 5:** Regression coefficients for working memory index in patients.

	**Patients**			**Controls**		
**Variable**	**Unstandardised** **b**	**Standardized** **B**	***p***	**Unstandardised b**	**Standardized B**	***p***
Subcortical volume	0.001	0.201	0.103	−0.001	−0.373	0.086
Hemoglobin	0.143	0.148	0.119	0.654	0.416	0.111
SpO_2_	0.290	0.057	0.538	3.254	0.349	0.063
Sex	−7.007	–**0.255**	0.027	−9.352	−0.350	0.149
Age	−0.303	–**0.198**	0.044	−0.282	−0.192	0.323
SES	0.461	0.073	0.428	−2.034	–**0.321**	**0.041**
ICV	2.902^E − 6^	0.027	0.845	3.979E-5	0.398	0.116
Hydroxyurea use	−4.563	−0.157	0.385	-	-	-

Adjusted *R*^2^, 0.069; SpO_2_, oxygen saturation from pulse oximetry; SES, socioeconomic status; ICV, intracranial volume.

Bold values signify statistical significance.

Separate multiple linear regression models for males and females with SCA revealed that WMV predicted PSI (Table 6) and total subcortical volume predicted WMI (Table 7) in males only. Age predicted PSI in both sexes (Table 6) and predicted WMI in females (Table 7). SES independently predicted PSI in males (Table 6) but did not predict WMI in either sex (Table 7). In male patients, individual subcortical regions predicting WMI were statistically significant for left and right thalamus only (Table 8).

**Table 6:** Regression coefficients for processing speed index in male and female patients.

**Variables**	**Males**			**Females**		
	**Unstandardised** **b**	**Standardized** **B**	**Significance**	**Unstandardised** **b**	**Standardized** **B**	**Significance**
WMV	0.000	**0.424**	0.019	3.076E-5	0.110	0.619
Hemoglobin	0.194	0.215	0.10	0.046	0.051	0.734
SpO_2_	−0.245	−0.047	0.708	0.709	0.169	0.238
Age	−0.617	–**0.402**	0.003	−0.522	–**0.314**	**0.044**
SES	1.572	**0.279**	0.03	−0.557	−0.093	0.543
ICV	−2.916E-5	−0.238	0.184	−2.892E-5	−0.227	0.906

Males: Adjusted *R*^2^ = 0.207; Females: Adjusted *R*^2^ = 0.032; WMV, white matter volume; SpO_2_, oxygen saturation from pulse oximetry; SES, socioeconomic status; ICV, intracranial volume.

Bold values signify statistical significance.

**Table 7:** Regression coefficients for working memory index in male and female patients.

**Variable**	**Males**		**Significance**	**Females**		**Significance**
	**Unstandardised** **b**	**Standardized** **B**		**Unstandardised** **b**	**Standardized** **B**	
Subcortical volume	0.001	**0.355** ^**a**^	0.025	0.000	−0.076	0.686
Hemoglobin	0.224	0.299	0.092	0.017	0.017	0.908
SpO_2_	−0.026	−0.005	0.971	0.466	0.101	0.470
Age	−0.266	−0.160	0.262	−0.794	–**0.315**	**0.026**
SES	0.150	0.025	0.851	0.520	0.079	0.590
ICV	4.300E-6	0.032	0.834	6.147E-6	0.048	0.800

Males: Adjusted *R*^2^, 0.126; Females: Adjusted *R*^2^, 0.000.

SpO_2_, oxygen saturation from pulse oximetry; SES, socioeconomic status; ICV, intracranial volume.

**Table 8:** Regression for working memory index for individual subcortical regions for male patients.

**Subcortical region**	**Adjusted *R*^2^**	**Estimated mean volume (mm^3^)**	**Unstandardised b**	**Standardized beta**	***p***
Left caudate	0.090	3808.040	0.007	0.247	0.061
Right putamen	0.045	5891.307	0.001	0.064	0.614
Left thalamus	**0.117**	8110.063	0.005	0.293^a^	**0.019**
Left putamen	0.046	6100.057	0.001	0.068	0.602
Left pallidum	0.065	1474.678	0.008	0.159	0.200
Left accumbens area	0.045	654.352	0.006	0.059	0.646
Left hippocampus	0.068	4028.603	0.007	0.160	0.175
Left amygdala	0.044	1704.197	−0.003	−0.045	0.713
Right thalamus	**0.130**	7088.522	0.007	0.305^a^	**0.011**
Right caudate	0.043	3861.283	0.001	0.039	0.766
Right pallidum	0.060	1598.027	0.010	0.146	0.255
Right accumbens area	0.094	665.590	0.029	0.231	0.053
Right hippocampus	0.064	4114.112	0.007	0.148	0.209
Right amygdala	0.042	1815.090	0.001	0.014	0.912

### 3.4. Developmental trajectory analysis

**Figure 2:**
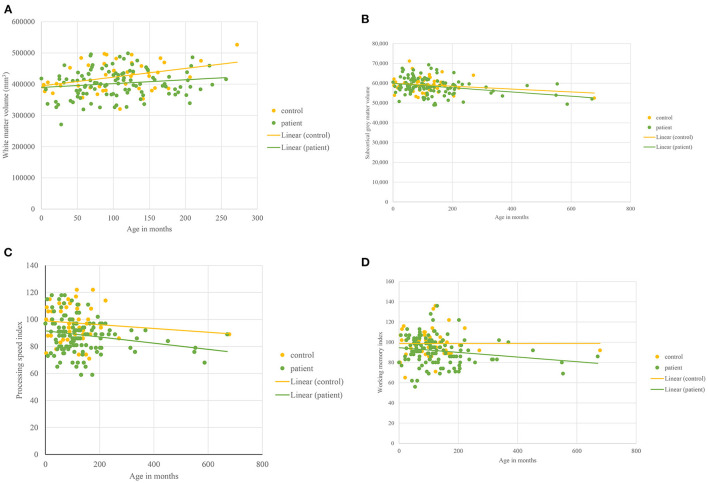
Developmental trajectories. For all figures, 0 denotes 8 years of age as it represents the earliest age at which data were collected. **(A)** White matter volume plotted against Age in patients with sickle cell disease and controls aged <30 years. **(B)** Total subcortical volume plotted against Age in patients with sickle cell disease and controls. **(C)** Processing speed index plotted against Age in patients with sickle cell disease and controls. **(D)** Working memory Index plotted against Age in patients with sickle cell disease and controls.

Developmental trajectories for WMV, PSI, total subcortical volume, and WMI are shown in Figure 2 and were compared between patients and controls (Table 9). Developmental trajectories are only valid if age significantly predicts variables in patients + controls or in the control group alone.

**Table 9:** Developmental trajectories.

**Variable**	**Slope: Volume/cognitive variable** = **(Intercept for group)** + **(Age** ^*^gradient)		**Delay at onset**	***p***	**Rate of development for patients**	***p***
	**Patients**	**Controls**				
WMV (mm^3^)	34082.693 + age ^*^ 169.871	−952.155 + age ^*^ 283.435	−35034.884	0.538	0.59	0.186
Subcortical Volume (mm^3^)	31336.313 + age ^*^−10.827	31390.132 + age ^*^ 2.27	53.819	0.627	−4.76	0.181
PSI	91.583 + age ^*^−0.023	98.947 + age ^*^−0.014	**7.36** ^a^	0.023	1.64	0.667
WMI	94.655 + age ^*^−0.023	96.687 + age ^*^ 0.022	2.03	0.993	−1.04	0.156

WMV, white mater volumes; PSI, processing speed index; WMI, working memory index.

Delay at onset, Intercept controls–intercept patient, Rate of development for patient, gradient patients/gradient controls.

a *p* < 0.001.

Bold values signify statistical significance.

Age predicted WMV (significantly) (*p* < 0.001) in the whole group (patients + controls). WMV increased for both patients and controls, and the rate did not differ between patients and controls nor were they significantly different at onset (Figure 2A, Table 9).

In patients, but not controls, age significantly predicted decline in total subcortical volume (*p* = .027). Subcortical gray matter volume decreased with age (Figure 2B, Table 9) in both patients and controls with no significant difference in onset or rate of decline.

Age predicted PSI (trend level) (*p* = 0.07) in the whole group (patients + controls). At onset (8.02 years), PSI (*p* < 0.001) was significantly lower in patients as compared to controls (Figure 2C, Table 9) with PSI declining with age in patients and controls (Figure 2C) but with no significant difference in rate (Table 9).

In patients but not controls, age significantly predicted decline in WMI (*p* = 0.043). In the trajectory analysis, WMI was maintained in controls and declined in patients (Figure 2D, Table 9) but the difference was not statistically significant.

## 4. Discussion

The purpose of this study was to investigate the effect of WMV on processing speed and the effect of total subcortical volume on working memory. We also aimed to compare cross-sectional developmental trajectories between patients with SCA and controls. In patients with SCA (male + female), WMV predicted processing speed at a trend level, providing some evidence supporting hypothesis 1. For hypothesis 2, there was weak evidence for an association between total subcortical volumes and WMI in patients and controls although neither was significant when males and females were included. However, these relationships were significant in male patients, supporting hypothesis 3. In line with the fourth hypothesis, some developmental trajectories differed between patients and controls. In patients, PSI was significantly delayed at onset (8.02 years).

**Figure 3:**
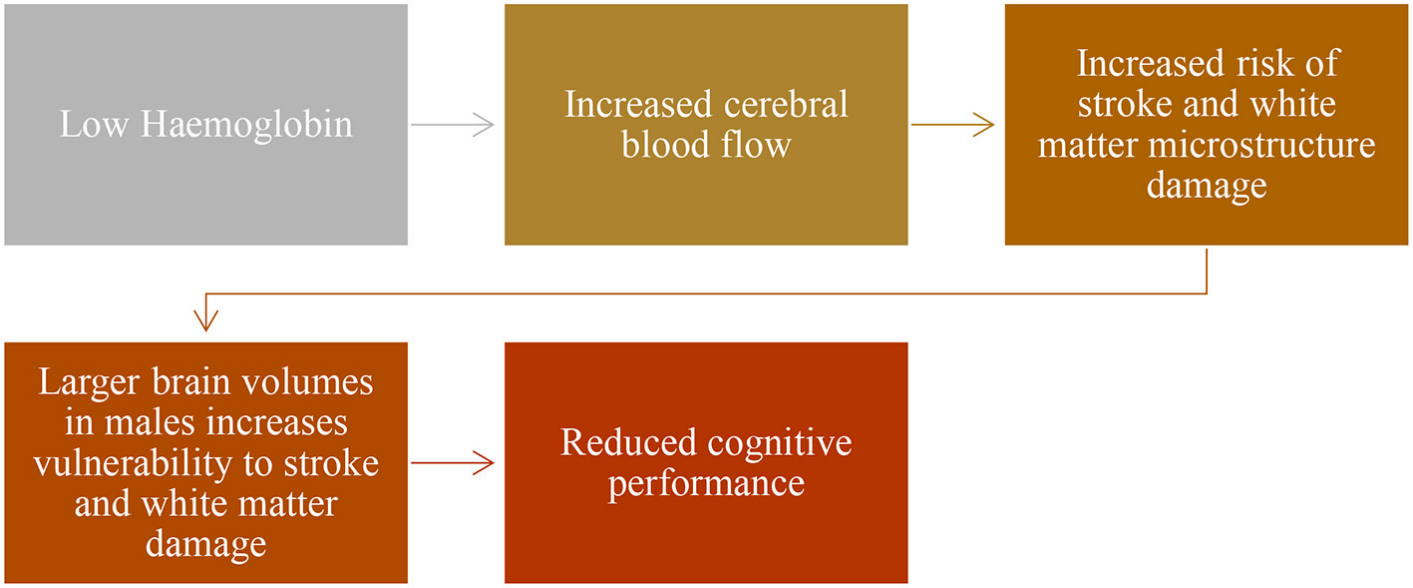
Beyond brain volume.

When both male and female patients with SCA were included, WMV was associated with processing speed at trend level. When the analysis was conducted only in male patients, this association was significant. We also found that in a model including age and sex, hemoglobin was independently associated with processing speed in patients. As processing speed appears compromised in childhood, cerebral hemodynamic compensation for anemia in early life may affect the development of white matter in patients, particularly in males, with a secondary effect on processing speed. Previous literature suggests that hemoglobin and cerebral blood flow velocity are associated with neurodevelopmental performance in 9-month-old infants with SCA (32). During the pre-school years, hemoglobin is lower in patients as compared to controls (32). Processing speed at this age is 1.5 SD below the mean (6). Furthermore, reduced PSI is associated with white matter microstructural damage in watershed areas in patients with SCA (33). It is likely that lower hemoglobin is compensated for by higher cerebral blood flow and higher oxygen extraction fraction (4) which may increase the risk for infarction (12). In males, larger brain volumes (34) may be associated with greater need for cerebral blood flow increasing risk of infarction (12) and white matter microstructural damage associated with reduced PSI (32) (see Figure 3).

No studies have looked at the association between subcortical volumes and working memory in children with SCA, making our contribution to the literature important. Previous studies have found an association between subcortical volumes and working memory in adults with SCA aged 19–55 years (9). Our results provide only weak evidence, which was not statistically significant, to support this finding. However, there was only one older control, and our study was underpowered to exclude the association over the wide age range. We included pediatric patients with SCA alongside adult patients which may nullify the effect of subtle neurodevelopmental differences present in adult patients with SCA. In addition, working memory is a complex phenomenon using brain regions including the frontal lobe, parietal cortex, subcortical regions, and the cerebellum (35). Subcortical regions have smaller volumes in pediatric populations with SCA (16). Working memory in the SCA population may be affected by compensatory function in other brain regions. In this study, working memory showed decline with age in patients with SCA only, likely due to the effect of anemia on brain structure and function (36–38).

Our study compared regional brain volume trajectories across a wide age range and found similar trajectories in the controls to previously published data (31) for WMV and total subcortical gray matter volume. We compared WMV in ages 8–30 years and subcortical volumes in patients with SCA and controls aged 8–64 years. WMV did not differ between patients and controls at 8 years nor did the rate of development significantly differ between patients and controls. However, age significantly predicted WMV growth in both groups. Similar findings have been reported by Steen et al. (19). We suspect that lower white matter density (39) and microstructural abnormalities (13) alongside cerebral hemodynamic mechanisms (4) are a better predictor of cognition in SCA patients as compared to WMV. Total subcortical volumes were significantly negatively predicted by age, suggesting reducing volumes with age. Moreover, at onset, SCA patients tend to have larger volumes compared to controls. However, this difference is not significant in our participant group. Previous studies indicate that children with SCA tend to display subtle neurodevelopmental delay in total gray matter volume growth related to SCA pathology (9, 34).

### 4.1. Limitations

We did not examine the effect of SCI in the patient or control group. Although literature suggests that cognition and brain growth is affected in the SCA population regardless of the presence of SCI, patients with SCI may be more likely to have severe disease (2). Evaluating the effect of SCI on developmental trajectories is an important next step. Additionally, our study did not plot separate developmental trajectories for males and females. A previous study on brain volume growth has demonstrated that there is no effect of sex on brain volume trajectories after controlling for total brain volume (34). However, this effect is yet to be evaluated for cognitive variables. Our study is also limited by our use of cross-sectional data. Although it is possible to make inferences about development using cross-sectional data, this will always be inferior to interrogating longitudinal data. Developmental trajectories may also be non-linear, while we only considered linear trajectories. Looking at curve estimations should be considered in future research. We were underpowered to exclude the possibility that subcortical volumes predict working memory. Further research in larger populations should investigate other brain regions implicated in working memory which may be involved in compensatory functions in children with SCA. Additionally, we used MNI template space to process our images. MNI template is mainly based on non-Hispanic white population which may affect image processing results. Further research may consider creating and using template space adapted for non-white populations. We were not able to establish a link between cognitive end points and use of hydroxyurea in patients. We believe this is due to inconsistent use of hydroxyurea in our cohort. Hence, adding measures of compliance in future studies should be considered. In addition, randomized controlled trials of new treatments for SCA should consider including cognitive and MRI endpoints to investigate their effects on the brain.

## 5. Conclusions

Through our study, we have contributed to the current literature on SCA pathology. Our findings suggest an association between white matter volume and processing speed in male patients with SCA. They also suggest that developmental trajectories may differ between patients with SCA and controls. These findings contribute to current understanding of disease severity in SCA populations. Interventions targeting early life cerebrovascular and hemodynamic mechanisms might preserve cognitive function and contribute to improved quality of life in this population. Another important finding is that females appear to have protective mechanisms against disease severity. Appropriate timely intervention for males, including monitoring hemoglobin levels, should be considered as a target for intervention. The observation that anemia severity affects brain volumetric growth in the SCA population supports existing literature (12). Future research should explore the effect of SCI and sex on disease severity in the patients with SCA.

## Data availability statement

The raw data supporting the conclusions of this article will be made available by the authors, without undue reservation.

## Ethics statement

The studies involving human participants were reviewed and approved by West London and South Yorkshire Research Ethics Committees. Written informed consent to participate in this study was provided by the participants' legal guardian/next of kin.

## Author contributions

SH conceptualized the paper, completed the review, analyzed the data, and drafted the manuscript. MK and HS were involved in data collection and processing. SH and MK analyzed the data supervised by FK, AH, RM, and JC. RM, JC, HS, and AH contributed to the review and provided feedback on the manuscript. All authors provided feedback on the manuscript and read and approved the final version for submission.
